# Multiscale strategies to enhance plant resilience under climate change

**DOI:** 10.1093/jxb/erag283

**Published:** 2026-07-10

**Authors:** Monica Balsera, Juan Manuel Pérez-Ruiz, Antonio Jesús Serrato

**Affiliations:** Department of Abiotic Stress, Instituto Natural de Recursos Naturales y Agrobiología, Salamanca, Spain; Instituto de Bioquímica Vegetal y Fotosíntesis, Universidad de Sevilla-CSIC, Sevilla, Spain; Department of Stress, Development and Signaling in Plants, Estación Experimental del Zaidín (EEZ-CSIC), Granada, Spain

**Keywords:** Climate change, environmental stress, hormonal crosstalk, plant–microbe interactions, plant performance, plant resilience, predictive plant biology, redox signalling, synthetic biology, systems biology

**Climate change is no longer a distant projection but an ongoing process redefining the boundaries of plant survival and ecosystem stability. Rising global temperatures, increasing water scarcity, and the degradation of arable soil, often compounded by shifting biotic pressures, are converging to create complex and rapidly changing environmental scenarios. In these contexts, the impact of stressors is not merely additive but synergistic and unpredictable. The contributions gathered in this Special Issue address how plants respond to these challenges by aligning biological processes across molecular, physiological, and ecological dimensions. Rather than focusing on isolated pathways, these studies highlight the interconnected networks—ranging from redox communication to plant–microbe synergies—that allow plants to perceive, respond, and acclimate to a changing world.**

Plants are exposed to fluctuating conditions that require tight coordination of growth, development, and defence across both spatial and temporal scales. Growing evidence indicates that these processes cannot be fully understood through isolated pathways or single-stress frameworks. Instead, plant resilience emerges from regulatory networks that merge cellular, physiological, and ecological responses, linking molecular events to whole-plant performance (Lamers *et al.*, 2020; Zandalinas *et al.*, 2024). Such interactions rely on interconnected signalling and regulatory mechanisms that coordinate cellular responses under fluctuating environmental conditions and ultimately determine plant performance under field conditions (Foyer, 2026). Given that plants face multifactorial constraints, their natural responses often diverge from those predicted under single-stress laboratory conditions (Suzuki *et al.*, 2014). Furthermore, acclimation is a non-static process, continuously tuned by the intensity, duration, and sequence of cues experienced throughout plant development. This temporal dimension of stress responses, including processes such as priming and stress memory, is increasingly recognized as a critical determinant of plant resilience (Nair *et al.*, 2022; Charng *et al.*, 2023). Consequently, experimental approaches are also progressively moving towards more realistic environmental scenarios that better capture the complexity experienced by plants under natural conditions (Moshelion *et al*., 2024; Bazakos *et al.*, 2026). To address this complexity, advances in multi-omics integration, systems biology, and predictive modelling are aligning basic mechanisms with field performance, opening up new venues for understanding and engineering plant resilience (Kundu and Tanti, 2026).

Interest in these emerging questions motivated the workshop ‘Plants under Environmental Stress: Overcoming Current Climate Challenges’ held in Baeza (Jaén, Spain) in November 2024. This meeting brought together researchers across diverse areas of plant biology, from redox control and photosynthesis to plant–microbe interactions, stress acclimation, biotechnology, and ecosystem responses to climate change, fostering discussions on how mechanistic understanding can be translated into strategies for sustainability and productivity. Building on these discussions, this Special Issue brings together contributions focused on bridging molecular insights with plant performance under realistic environmental conditions, while also incorporating complementary perspectives from across the discipline. A central theme throughout these studies is the imperative to bridge different biological scales. Collectively, the works presented here illustrate how plant resilience is shaped by processes spanning intracellular crosstalk and metabolic fine-tuning to developmental plasticity, ecological interactions, and ecosystem-scale restoration.

## Redox signalling as an organizing principle

One of the most prominent themes emerging from this collection is the central role of redox biology as a framework for understanding signalling specificity and environmental responsiveness. Redox processes are no longer viewed merely as consequences of cellular damage; instead, they represent a fundamental language through which plants process information and fine-tune their responses.

This is particularly evident in the review by Jiménez *et al.* (2026), who examine how thioredoxins (Trxs) connect redox and calcium (Ca^2+^) transduction. In the early stages of stress perception, the reciprocal relationship between reactive oxygen species (ROS) bursts and Ca^2+^ transients creates a high-fidelity regulatory hub. Their work positions Trxs not just as antioxidants, but as master regulators capable of reversing oxidative thiol modifications on key Ca^2+^ transporters and sensors, thereby resetting the cellular state. A related yet distinct layer of thiol-based control is explored by Romero *et al*. (2026), who review hydrogen sulfide (H_2_S) signalling through persulfidation of specific oxidized cysteines in target proteins. Persulfidation represents a ubiquitous post-translational modification (PTM) that alters protein conformation and activity in response to gasotransmitter signals. Crucially, the discovery that the Trx system also governs depersulfidation suggests a highly fluid and reversible regulatory landscape where different sulfur-based modifications compete or cooperate to adjust enzyme functions under fluctuating conditions. Together, these studies reinforce the view that redox-dependent regulation operates as a central integrative layer linking environmental perception with downstream physiological responses across scales. However, stress perception alone is insufficient unless these signals are ultimately translated into coordinated physiological and developmental responses.

## Developmental plasticity through signalling integration

If redox sensors act as the primary trigger for environmental fluctuations, developmental plasticity represents the downstream execution that ensures long-term survival. Plants must constantly navigate the growth–defence trade-off, strategically allocating limited resources between biomass production and stress acclimation. Such adjustments emerge from the interplay of hormonal, redox, and epigenetic networks (Devireddy *et al.*, 2021).

The study by Rubio-Heras *et al*. (2026) exemplifies this unification by dissecting the interplay between nitric oxide (NO) and brassinosteroid (BR) pathways during post-germinative growth. By demonstrating how NO accumulation modulates endogenous BR levels and activity in an organ-specific manner, the authors transcend simplistic antagonistic models. Instead, they show that growth promotion or restraint is a context-dependent output of overlapping signalling gradients. Parallel to this, Sánchez-Vicente *et al*. (2026) explore the redox regulation of the transcription factor ABSCISIC INSENSITIVE 5 (ABI5) during the critical window of seed germination. Reversible oxidative modifications on ABI5 determine its stability and DNA-binding affinity, illustrating how a single molecular switch integrates environmental inputs to govern the success of a pivotal developmental transition.

## Organellar functions and the architecture of resilience

These developmental outputs ultimately depend on the capacity of organelles to sustain metabolic stability and coordinate cellular homeostasis under fluctuating environmental conditions (Sweetlove and Fernie, 2013; Gibbs, 2026). Within this framework, organelles have emerged as central regulatory hubs that link environmental cues to adjust gene expression, protein activity, and metabolism (Kmiecik *et al*., 2016; Crawford *et al*., 2018; Bittner *et al*., 2022). Processes such as the unfolded protein response and retrograde communication illustrate how acclimation emerges from the constant interplay between proteostasis and inter-organellar crosstalk (Afrin *et al*., 2020; Sevilla *et al*., 2023; Lee and Kim, 2024; Loudya *et al.*, 2024).

This perspective is reflected in the contribution of Sandalio *et al.* (2026), which addresses complementary aspects of organelle maintenance under heavy metal stress. Their findings emphasize that autophagy is not merely a passive degradation pathway but a sophisticated recycling mechanism that sequesters toxic ions and redistributes nutrients to sustain cellular homeostasis. Complementing this, Izquierdo *et al*. (2026) demonstrate that ethylene signalling can enhance mitochondrial stress tolerance independently of the classical ANAC017 pathway. This discovery challenges existing models of retrograde signalling hierarchies, suggesting a more decentralized and redundant network for organelle maintenance than previously envisaged.

## From symbiosis to ecosystem engineering

Plant performance is strongly influenced by interactions with other organisms, which increasingly appear as integral components of development and environmental responsiveness (Berg *et al*., 2016). In this issue, several contributions expand the study of plant–microbe interactions, surpassing conventional roles centred on nutrition. Armijo-Godoy *et al.* (2026) describe a non-canonical beneficial interaction between *Arabidopsis thaliana* and *Sinorhizobium meliloti* under nitrogen deficiency, displacing the traditional legume–rhizobium paradigm. Similarly, Mishra *et al.* (2026) demonstrate how plant growth-promoting *Pseudomonas* strains directly influence developmental programmes, such as tuberization signalling and root development in potato. This perspective is further deepened by Devesa-Aranguren *et al*. (2026), who argue that root–microbiome interactions involved in heat stress resilience should be examined through the lens of epigenetic regulation. Importantly, these studies highlight that plant resilience extends beyond the individual organism and emerges from dynamic interactions within biological communities. As climate change alters both biotic and abiotic conditions, resilience becomes increasingly dependent on the coordinated responses of plants and their associated microbiota. Furthermore, incorporating natural variation from wild species and locally adapted genotypes represents a critical, yet underexploited, resource for identifying traits associated with stress resilience.

At a broader scale, Martínez (2026) reviews how molecular interactions between plants and arthropod herbivores are altered by shifting climates, an area highly sensitive to specific environmental cues (Lin *et al*., 2023). Extending even further, Parsons *et al*. (2026) explore peat moss (*Sphagnum*) as a tool for ecosystem restoration and climate mitigation. Together, these contributions connect organismal responses with ecosystem-level processes, reinforcing the need to integrate multiple scales when assessing plant performance under climate change.

## From discovery to design

A defining trend within this Special Issue is the transition toward system-wide approaches that connect regulatory processes across biological scales. This shift from discovery-driven research toward precision engineering is exemplified by Meile *et al*. (2026), who develop a high-throughput heterologous expression platform for rapid multigene testing. Such systems provide scalable platforms for the reconstruction and functional analysis of complex pathways that remain difficult to resolve through conventional methods. In parallel, advances in systems biology and artificial intelligence are expanding our capacity to analyse complex datasets and predict plant responses. In this context, Santos-del Río *et al*. (2026) explore how computational frameworks facilitate the integration of multi-layered data to improve the prediction of plant responses to salinity. These advances open up the possibility of designing crops with enhanced resilience to combined stresses, such as drought, heat, and pathogen pressure, which more accurately reflect field conditions. Crucially, this design process must incorporate the temporal dimension of stress responses, ensuring that adaptive traits remain effective across developmental stages and environmental fluctuations.

## Conclusion

Collectively, the articles in this Special Issue illustrate that the next frontier in plant science lies not simply in bridging the gap between controlled mechanistic studies and the complexity of natural environments, but in defining the principles that make such integration possible. While reductionist approaches established a robust framework for understanding individual pathways, they often fail to capture the dynamic and combinatorial nature of stress conditions encountered in the field.

**Fig. 1.**
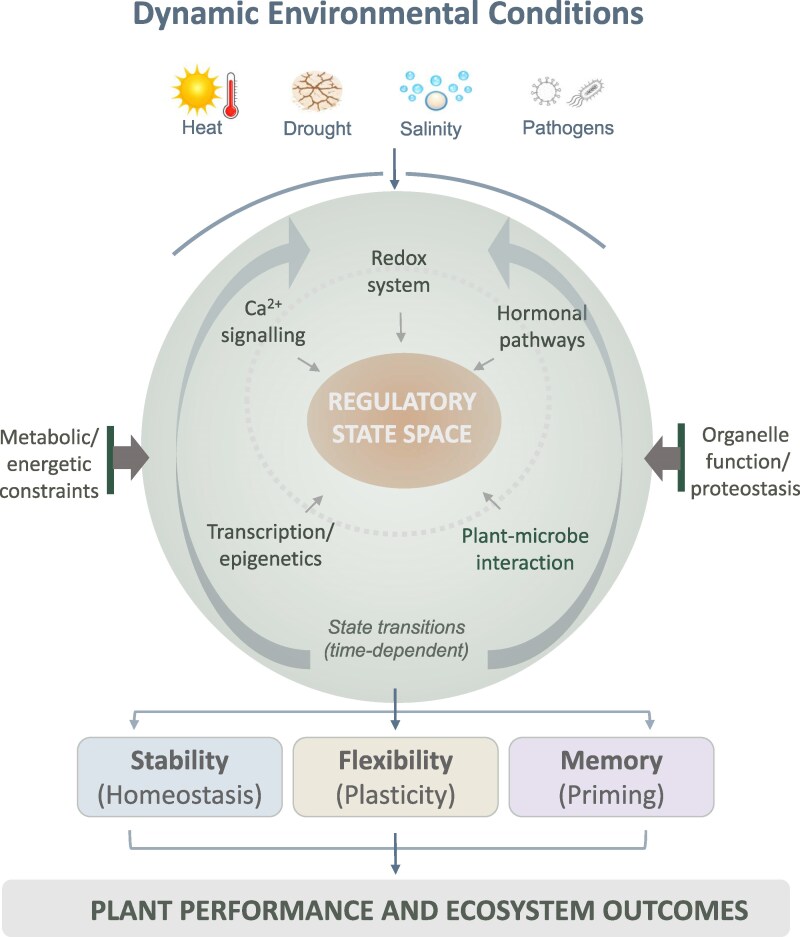
Multiscale integration of plant responses to dynamic environmental conditions. Plants experience fluctuating environmental conditions, including heat, drought, salinity, and biotic pressures, among others. Within this context, plant responses emerge from the coordinated interaction of multiple regulatory layers, such as calcium signalling, hormonal pathways, redox processes, transcriptional and epigenetic regulation, and plant–microbe interactions. These interconnected systems operate within metabolic and cellular constraints and define a dynamic regulatory space that continuously adjusts through time-dependent state transitions. The integration of these processes underlies key emergent properties of plant behaviour, including stability (homeostasis), flexibility (developmental plasticity), and memory (stress priming), which together determine plant performance and ecosystem-level outcomes.

Plant resilience may be better understood not as the mere outcome of multiscale integration, but as an emergent property of coordinated networks operating across molecular, cellular, and physiological levels. Within this framework, redox signalling represents one component within a broader network coordinating processes including signal transduction, hormonal crosstalk, transcriptional regulation, protein regulation and plant–microbe interactions. As illustrated in Fig. 1, plant responses emerge through the interplay of these layers, linking signalling dynamics with multiscale outputs such as physiological adjustments, developmental plasticity, stress acclimation, and resource allocation. Together, these processes shape plant performance and determine outcomes at agricultural and ecosystem levels. This view shifts the focus from static pathway interactions to system-level behaviour, where properties such as stability, plasticity, and stress memory arise from the coordinated action of complex biological networks.

Moving from a descriptive to a predictive discipline will thus require not only the integration of multi-omics data and advanced modelling approaches, but also the identification of the mechanisms shaping system behaviour across scales. Defining these mechanisms may bring us closer to understanding not only how plants respond to stress, but also how resilience can be strategically enhanced. Such a perspective will be essential for developing crops capable of maintaining performance under increasingly variable conditions, thereby helping to address the challenges posed by climate change and the need to sustain agricultural productivity and ecosystem stability under increasingly variable conditions.
